# Impact of Maternal Obesity and Diabetes on Long-Term Health of the Offspring

**DOI:** 10.1155/2011/163438

**Published:** 2011-12-18

**Authors:** Christine Maric-Bilkan, Michael Symonds, Susan Ozanne, Barbara T. Alexander

**Affiliations:** ^1^Department of Physiology and Biophysics, University of Mississippi Medical Center, 2500 North State Street, Jackson, MS 39216, USA; ^2^Women's Health Research Center, University of Mississippi Medical Center, 2500 North State Street, Jackson, MS 39216, USA; ^3^Division of Child Health, School of Clinical Sciences, Queen's Medical Centre University Hospital, Nottingham NG7 2UH, UK; ^4^Institute of Metabolic Science, University of Cambridge, Cambridge CB2 3EG, UK

The initial observations of David Barker, popularly known as the “Barker hypothesis” or “developmental origins of health and disease,” show that being born with low birth weight, as a result of intrauterine growth restriction produced by maternal undernutrition, is associated with a number of chronic diseases later in life [1]. Subsequently, studies show that it is not just intrauterine growth restriction, but also exposure to any other adverse factor during fetal and/or early postnatal development that can increase susceptibility to a number of chronic diseases later in life including cardiovascular and renal disease, hypertension, type 2 diabetes, certain forms of cancer, osteoporosis, Parkinson's disease, dementia, and polycystic ovary syndrome [2–4]. 

Over 346 million people worldwide have diabetes, and according to the estimates of the World Health Organization, the prevalence of type 2 diabetes will double by the year 2030 [5]. Similarly, the prevalence of obesity has reached alarming levels. There are 1.5 billion adults, 20 years of age and older, who are overweight. Of those, 200 million men and nearly 300 million women are obese [6]. As a result of this growing prevalence of type 2 diabetes and obesity, more and more women of child-bearing age are either obese and/or diabetic during pregnancy. Given that maternal health has a significant impact on the long-term health of the offspring, it is clear that both type 2 diabetes and obesity are not only a health concern for the mother, but also a growing concern for the generations to come. Thus, the importance of examining the impact of maternal overnutrition and diabetes on the long-term health of the offspring is paramount.

Several experimental studies report a relationship between maternal overnutrition and health of the offspring in adulthood. Specifically, maternal body weight, overnutrition, or a high fat consumption during pregnancy is linked to the development of elements of the metabolic syndrome, cardiovascular and renal disease, hypertension, and cerebral dysfunction as well as type 2 diabetes and obesity themselves later in the life of the offspring [7–12]. However, the mechanisms by which type 2 diabetes and obesity lead to the development of chronic disease later in life remain unknown. The purpose of this issue is to compile and provide a forum for the discussion of the latest data on the impact of maternal overnutrition and diabetes on the long-term (metabolic) health of the offspring.

The current issue presents 5 clinical research papers as well as 7 review articles. The following is a summary of major points and findings presented in these papers.

1. In overweight/obese mothers, greater % kcal from sweets early in pregnancy is the strongest, independent predictor of higher weight for gestational age at birth as well as after 6 months and higher odds of fetal macrosomia, suggesting that mothers' eating behaviors during pregnancy may have a lasting effect on child weight.
2. In nondiabetic women, maternal glucose levels correlate with the extent and distribution of fetal adiposity and birth weight.
3. In the Jerusalem perinatal study (a cohort of over 92,000 births), offspring of mothers with gestational diabetes have higher body mass index and systolic and diastolic blood pressure at 17 years of age compared with offspring of mothers with no gestational diabetes. These data suggest an association between maternal glycemia and cardiometabolic outcomes in the offspring.
4. Interestingly, no association between cardiac function in offspring of type 1 diabetic mothers at 7-8 years of age and maternal glycemic control was found. Whether the cardiac phenotype takes longer to develop in this cohort may need to be examined.
5. At the age of 3 years, offspring of type 1 diabetic mothers were characterized by a delay in cortical evoked responses in both visual and somatosensory systems, suggesting a potential association between maternal glycemia and brain maturation in the offspring.
6. The 7 review articles all provide a comprehensive summary of some of the mechanisms underlying the developmental programming of metabolic syndrome, cardiovascular disease, and obesity later in the life of the offspring. Interestingly, similar mechanisms seem to contribute to different phenotypic outcomes regardless of what the insult was during early development. Specific emphasis is given to the importance of epigenetic changes induced by the maternal environment in predicting later adversity.

In summary, this special issue highlights the fact that maternal health plays a significant role in determining as well as predicating the health of the offspring later in their life. Future studies are warranted to specifically examine the mechanisms by which the perturbation of the *in utero* environment may translate into a health risk in the offspring.

*Christine Maric-Bilkan* *Christine Maric-Bilkan* *Michael Symonds* *Michael Symonds* *Susan Ozanne* *Susan Ozanne* *Barbara T. Alexander* *Barbara T. Alexander*
